# DA‐7503, a novel tau aggregation inhibitor, ameliorates tau pathology and reduces CSF tau in transgenic mouse models of Alzheimer’s disease and primary tauopathies

**DOI:** 10.1002/alz.084642

**Published:** 2025-01-09

**Authors:** Min Jung Lee, Song‐hyen Choi, Jun‐Hwan Moon, Hyangsoo Lee, Eunjin Kim, Dae Young Lee, Sungsu Lim, Yun Kyung Kim, Ae Nim Pae

**Affiliations:** ^1^ Dong‐A ST Research Headquarter, Yongin Korea, Republic of (South); ^2^ Korea Institute of Science and Technology, Seoul Korea, Republic of (South)

## Abstract

**Background:**

Elevation of cerebrospinal fluid (CSF) tau is a feature of Alzheimer’s disease (AD) and is being explored as a biomarker of AD and other tauopathies. The aim of this study was to elucidate the *in vivo* effects of DA‐7503, a potent and selective tau aggregation inhibitor, and its pharmacodynamics on CSF tau in transgenic mouse models of Alzheimer’s disease and primary tauopathies.

**Method:**

TauP301L‐BiFC mice expressing full‐length human tau with the P301L mutation were orally administrated with DA‐7503 for 1 month. CSF, plasma, and brain homogenates were collected at 4 time points from the last dosing. Total tau in CSF was measured using an AlphaLISA, and the concentrations of DA‐7503 in CSF and plasma by time were determined using LC‐MS/MS. To further evaluate the effectiveness of DA‐7503, neurobehavior and tau pathology were investigated after oral administration of DA‐7503 in TauP301L‐BiFC mice. The effect of DA‐7503 on tau pathology was also determined in human Tau (hTau) transgenic mice expressing all six isoforms of human tau combined with a knockout of murine tau.

**Result:**

In a TauP301L‐BiFC AD mouse model, DA‐7503 showed not only a significant reversal of memory and recognition deficits but also an attenuation of tau aggregation and phosphorylation in the cortex and hippocampus. In these mice, DA‐7503 caused a significant reduction of CSF tau in a drug concentration‐dependent manner. DA‐7503 also reduced phosphoSer202‐tau in the brain cortex of hTau transgenic mice.

**Conclusion:**

DA‐7503 restores the impairments of memory and recognition, and ameliorates the aggregation and hyperphosphorylation of tau in transgenic mouse models of AD and other tauopathies. The inverse correlation between total Tau in CSF and DA‐7503 exposure in plasma suggests that DA‐7503 induces an increase of tau clearance from the brain and CSF tau may be explored as a biomarker in clinical development of DA‐7503. Our findings indicate DA‐7503 is a promising agent for the treatment of AD and primary tauopathies.

